# Antileukotriene Reverts the Early Effects of Inflammatory Response of Distal Parenchyma in Experimental Chronic Allergic Inflammation

**DOI:** 10.1155/2013/523761

**Published:** 2013-09-15

**Authors:** Nathália Brandão Gobbato, Flávia Castro Ribas de Souza, Stella Bruna Napolitano Fumagalli, Fernanda Degobbi Tenório Quirino dos Santos Lopes, Carla Máximo Prado, Milton Arruda Martins, Iolanda de Fátima Lopes Calvo Tibério, Edna Aparecida Leick

**Affiliations:** ^1^Medicine Department, School of Medicine, University of São Paulo(USP), 01246-903 São Paulo, SP, Brazil; ^2^Biological Science Department, Universidade Federal de São Paulo(UNIFESP), 09972-270 Diadema, SP, Brazil

## Abstract

*Aims*. Compare the effects of montelukast or dexamethasone in distal lung parenchyma and airway walls of guinea pigs (GP) with chronic allergic inflammation. *Methods*. GP have inhaled ovalbumin (OVA group-2x/week/4weeks). After the 4th inhalation, GP were treated with montelukast or dexamethasone. After 72 hours of the 7th inhalation, GP were anesthetised, and lungs were removed and submitted to histopathological evaluation. *Results*. Montelukast and dexamethasone treatments reduced the number of eosinophils in airway wall and distal lung parenchyma compared to OVA group (*P* < 0.05). On distal parenchyma, both treatments were effective in reducing RANTES, NF-**κ**B, and fibronectin positive cells compared to OVA group (*P* < 0.001). Montelukast was more effective in reducing eotaxin positive cells on distal parenchyma compared to dexamethasone treatment (*P* < 0.001), while there was a more expressive reduction of IGF-I positive cells in OVA-D group (*P* < 0.001). On airway walls, montelukast and dexamethasone were effective in reducing IGF-I, RANTES, and fibronectin positive cells compared to OVA group (*P* < 0.05). Dexamethasone was more effective in reducing the number of eotaxin and NF-**κ**B positive cells than Montelukast (*P* < 0.05). *Conclusions*. In this animal model, both treatments were effective in modulating allergic inflammation and remodeling distal lung parenchyma and airway wall, contributing to a better control of the inflammatory response.

## 1. Introduction

Asthma plays an important role as a major cause of mortality and morbidity all over the world. Data from the World Health Organization estimates that 100 millions to 150 millions people worldwide have a diagnosis of asthma [1], and around 180.000 people around the world die, every year, from this disease. Moreover, asthma affects people in school age and economically active people, having a high social and economic impact with lost productivity and reduced participation in family life. Therefore, it is considered a public health problem with high cost medications and hospitalizations of patients, enhancing the importance of continuing studying asthma.

Bronchial asthma is a chronic inflammatory disorder involving chronic airway inflammation, tissue remodeling, and declined airway function. The asthma physiopathology involves mediators and many cells, mainly eosinophils which plays important role through the release of specific mediators [2], and the degree of eosinophilia has been related with the disease severity in some asthmatic patients [3].

Eosinophil recruitment at the airway tissue is considered a multifaceted mechanism which involves chemokines such as eotaxins and RANTES. Besides that, a wide array of growth factors can be involved in airway inflammation, including insulin-like growth factor-1 (IGF-I) which is upregulated during this event [4], contributing with the remodeling process [5]. Furthermore, we may also consider the extracellular matrix (ECM) proteins, as fibronectin for example. Increased deposition of fibronectin and collagen into the subepithelial space of the airways is observed in all forms of asthma and appears very early in the disease [6].

Much of the subjacent inflammation of asthma can be mediated by the transcription nuclear factor-kappa B (NF-*κ*B), which is involved in the regulation of many of the inflammatory proteins that are expressed in asthmatic airways [7].

The importance of distal lung responses to the global pulmonary alterations enhancing asthma symptoms, particularly on severe asthma, has been recently addressed [8–10]. The inflammation process in the peripheral airways has a highaffinity to cysteinyl leukotriene-1 receptors (CysLT_1_Rs), and it is known that inhaled therapies are insufficient to achieve the small airways [11]. It is also important to highlight that important features of remodeling seem to occur mainly in the small airways [12], emphasizing the importance of targeting peripheral inflammation to achieve the disease control.

Our group had previously shown that in the ovalbumin-induced guinea pig asthma model, repeated allergen exposure leads to chronic allergic airway and distal lung parenchyma inflammation, characterized by an influx of eosinophils, mast cells, and allergen-specific T cells, a Th2-type cytokine pattern and airway and tissue remodeling [8, 13, 14].

Corticosteroids are the first-line therapy to threat asthma patients and were considered the most important drugs to improve hyperreactivity and bronchial hyperresponsiveness. However, antileukotrienes may also be considered to control asthma patients since cysteinyl leukotrienes (CysLTs) have been known to play an important role in bronchoconstriction and airway inflammation. CysLTs are synthesized from arachidonic acid and most of their actions are mediated by the CysLT_1_Rs, a G protein-coupled receptor. CysLTs have many pulmonary actions, including human airway smooth muscle contraction, chemotaxis, mucous secretion, smooth muscle proliferation, and increase in vascular permeability [15–18].

Clinical trials with anti-leukotriene drugs in mild and moderate persistent asthmatics have shown improvement in pulmonary function, symptoms, and need for rescue medications and a reduction in asthma exacerbations [19, 20]. It has also been shown that antileukotrienes reduce sputum and mucosal eosinophils in subjects with asthma [21]. However, recent long duration trials have evaluated the impact of antileukotrienes in comparison to glucocorticoids and showed that symptoms, spirometry, *β*_2_-agonist use, and also quality of life were improved to a greater extent with glucocorticoids [22, 23].

Using the same experimental model described in the present study, we observed that both montelukast or dexamethasone treatments diminished collagen fiber content, metallopeptidase inhibitor-1 (TIMP-1), matrix metallopeptidase-9 (MMP-9) and transforming growth factor (TGF-*β*) in distal lung parenchyma. Nonetheless, concerning elastic fiber content, dexamethasone's treatment did not reduce this response [24]. These foundings are in agreement with the results observed by Goleva et al. [25], in the evaluation of asthmatic patients resistant or not to corticosteroids.

Concerning these aspects, we considered pertinent to evaluate the effects of corticosteroids (dexamethasone) and antileukotrienes (montelukast sodium) in the eosinophilic inflammation and remodeling process modulated by fibronectin and IGF-I in an animal model of chronic allergic inflammation, studying both compartments, airway walls, and distal lung parenchyma.

## 2. Material and Methods

Guinea pigs (GP) were maintained in an animal facility with a 12-hour light/dark cycle and fed water and chow *ad libitum*. GP received humane care in compliance with the “Guide for care and use of laboratory animals” [26], and all experiments described in this study were previously approved by the Institutional Review Board of the University of São Paulo (São Paulo, Brazil/CAPpesq number 0276/09).

### 2.1. Experimental Groups

Four groups of GP were studied: (a) saline group (SAL, *n* = 8); (b) ovalbumin-sensitized GP (OVA, *n* = 8); (c) ovalbumin-sensitized GP treated with montelukast (OVA-M, *n* = 8); (d) ovalbumin-sensitized GP treated with dexamethasone (OVA-D, *n* = 8).

### 2.2. Induction of Chronic Pulmonary Allergic Inflammation

Male Hartley GP, weighing 300–400 g, were placed in a plexiglass box (30 × 15 × 20 cm) coupled to an ultrasonic nebulizer (Soniclear, São Paulo, Brazil). A solution of ovalbumin (OVA, Grade V, Sigma Chemical Co., Saint Louis, MO, USA) diluted in 0.9% NaCl (sterile saline solution) was prepared. The animals received seven inhalations during four weeks with increasing concentrations of OVA (1~5 mg/mL) in order to avoid tolerance. Control animals received aerosolized normal saline. The solution was continuously aerosolized into the environment until respiratory distress occurred, or until 15 minutes had elapsed, as previously described [13, 14, 27]. The observer who made the decision to withdraw the guinea pig from the inhalation box was blinded to the treatment status of the animal.

### 2.3. Montelukast and Dexamethasone Treatments

In order to avoid interference with sensitization, only after twenty-four hours of the fourth inhalation guinea pigs started to receive either daily oral montelukast sodium 10 mg·kg^−1^ (four hours before inhalations of ovalbumin and at the same hour on the other days) or dexamethasone (5 mg·kg^−1^/day i.p.) [13, 28].

### 2.4. Morphometric Studies

After 4 weeks, guinea pigs were anesthetized with sodium pentobarbital (50 mg/kg intraperitoneally), tracheostomized, and ventilated at 60 breaths/min (Harvard Apparatus South Natick, MA). Ten minutes after OVA challenge, a positive end-expiratory pressure of 5 cm H_2_O was applied to the respiratory system, and the airways were occluded at the end of expiration. GP were exsanguinated via the abdominal aorta, and lungs were removed *en bloc*.

Lungs were fixed with buffered 4% formaldehyde. Sections representing peripheral areas were processed for paraffin embedding. Histological sections of 3–5 *μ*m in thickness were stained and evaluated by persons blinded to the protocol design. Morphometric analysis was performed with a light microscope (Nikon, E200, City, Japan), with an integrating eyepiece (composed of 100 points and 50 lines) using a point-counting technique [13, 14, 29].

Three to five airways randomly selected from each lung slide were focused at a magnification of ×1,000. The number of eosinophils and positive cells for IGF-I, eotaxin, RANTES, fibronectin, and NF-*κ*B was determined as numbers of points of the integrating eyepiece falling on areas of cells or positive cells in three randomly selected areas of each airway wall divided by the number pointing in the airway wall area (10^4^ *μ*m^2^) [13, 14, 29].

To measure the number of positive cells in distal lung parenchyma, we counted in 10 fields per slide the number of eosinophils and positive cells for IGF-I, eotaxin, RANTES, fibronectin, and NF-*κ*B in each field divided by alveolar tissue area. Measurements were expressed as cells/10^4^ *μ*m^2^, at ×1,000 magnification (10^4^ *μ*m^2^), divided by the number pointing in the parenchyma area (10^4^ *μ*m^2^) [29, 30].

### 2.5. Measurement of Eosinophil Density

Five *μ*m thick slides of lung were stained with Luna [13, 31]. We analyzed the density of eosinophils within the alveolar septa and in airway walls as described above. Measurements were expressed as cells/10^4^ *μ*m^2^.

### 2.6. Immunohistochemistry

Immunohistochemistry was performed with anti-IGF-I (1:75; Santa Cruz—Sc 9013), anti-NF-*κ*B (1:50; Santa Cruz—Sc 109), anti-fibronectin (1:400; DAKO 0245), anti-eotaxin (1:100; Santa Cruz—Sc 6181), and anti-RANTES (1:400; Santa Cruz—Sc 1410) antibodies, by peroxidase method [24, 29].

Using a 100-point grid (area: 10^4^ *μ*m^2^at ×1,000 magnification), the number of the positive cells for IGF-I, eotaxin, RANTES, fibronectin, and NF-*κ*B was counted as mentioned above in Section 2.4.

### 2.7. Data Analysis

Values are expressed as mean ± standard error (SEM). Statistical analysis was performed using *SigmaStat* software (SPSS Inc, Chicago, IL, USA). Data were evaluated by one-way analysis of variance (ANOVA) and multiple comparisons were made using *Holm-Sidak* method. The *P* value < 0.05 was considered significant [32].

## 3. Results

### 3.1. Measurements of Eosinophils Density

**Figure 1:**
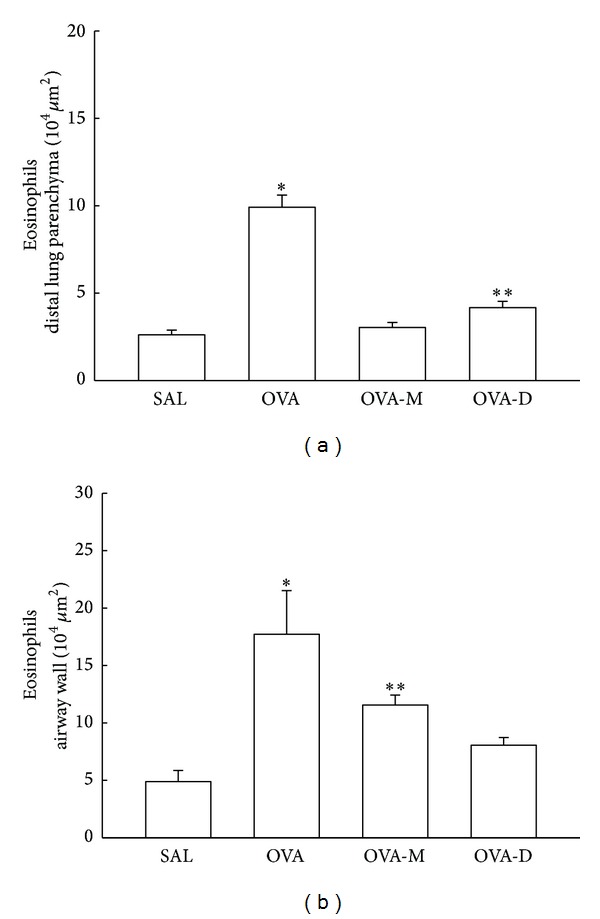
(a) Mean and SEM values of eosinophil in distal lung of GP that previously inhaled normal saline or ovalbumin, and after the 4th inhalation, GP were treated with montelukast (OVA-M group) and dexamethasone (OVA-D group).*P < 0.05 compared to the other groups. **P < 0.05 compared to SAL group. (b) Mean and SEM values of eosinophil in airway wall of GP that previously inhaled normal saline or ovalbumin, and after the 4th inhalation, GP were treated with montelukast (OVA-M group) and dexamethasone (OVA-D group). *P < 0.05 compared to the other groups. **P < 0.05 compared to SAL group.

Figure 1(a) presents the mean and SEM values of eosinophilic recruitment in distal lung parenchyma. We observed a significant increase in the number of eosinophils (cells/10^4^ *μ*m^2^) in OVA group (9.91 ± 0.70) compared to control (SAL group: 2.62 ± 0.26). There was a decrease of eosinophils in OVA-M (3.03 ± 0.28) and OVA-D (4.17 ± 0.36) compared to OVA (*P* < 0.05). There were no significant differences between OVA-M group and OVA-D group.

Figure 1(b) presents the mean and SEM values of eosinophilic recruitment in airway walls. We observed a significant increase in the number of eosinophils (cells/10^4^ *μ*m^2^) in OVA group (17.72 ± 3.79) compared to control (SAL group: 4.89 ± 0.97). There was a decrease of eosinophils in OVA-M (11.55 ± 0.87) and OVA-D (8.05 ± 0.67) compared to OVA(*P* < 0.05). There were no significant differences between OVA-M group and OVA-D group.

### 3.2. Measurements of Eotaxin Expression

**Figure 2:**
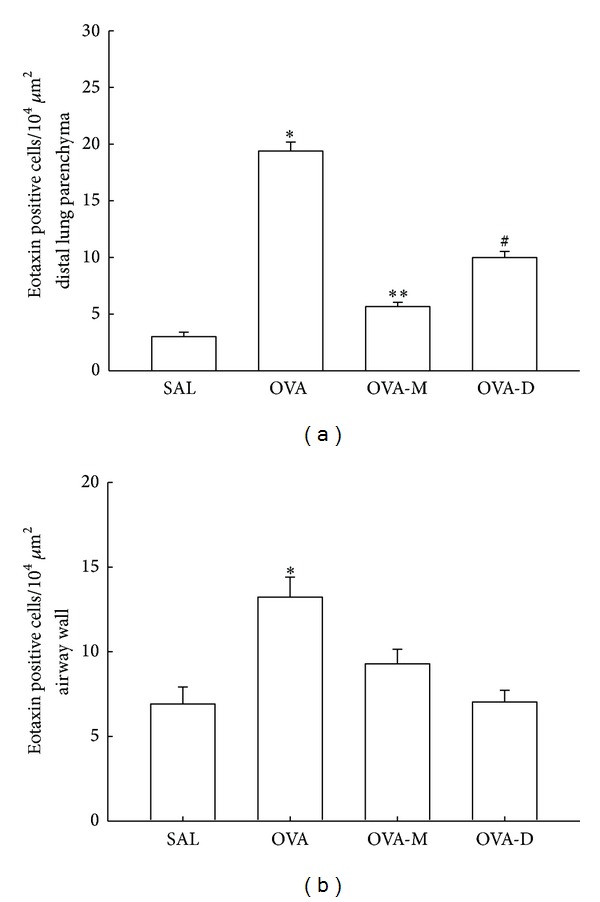
(a) Mean and SEM values of eotaxin positive cells in distal lung of GP that previously inhaled normal saline or ovalbumin, and after the 4th inhalation, GP were treated with montelukast (OVA-M group) and dexamethasone (OVA-D group). *P < 0.001 compared to the other groups. **P < 0.001 compared to SAL and OVA-D groups. ^#^ *P* < 0.001 compared to SAL group. (b) Mean and SEM values of Eotaxin positive cells in airway wall of GP that previously inhaled normal saline or ovalbumin, and after the 4th inhalation, GP were treated with montelukast (OVA-M group) and dexamethasone (OVA-D group). *P < 0.001 compared to SAL, OVA-M and OVA-D groups.

Considering eotaxin expression in inflammatory cells (Figure 2(a)), we observed a significant increase in the number of eotaxin positive cells (10^4^ *μ*m^2^) in distal lung parenchyma in OVA group (19.38 ± 0.79) compared to control (SAL group: 3.00 ± 0.39, *P* < 0.001). There was a decrease of eotaxin positive cells in OVA-M (5.65 ± 0.38) and OVA-D (9.9 ± 0.54) compared to OVA(*P* < 0.001). There was a significant reduction of eotaxin positive cells in OVA-M compared to OVA-D (*P* < 0.001).

Figure 2(b) shows eotaxin expression in inflammatory cells in airway walls. We observed a significant increase in the number of eotaxin positive cells (10^4^ *μ*m^2^) on airway walls in OVA group (13.23 ± 1.18) compared to control (SAL group: 6.92 ± 0.99, *P* < 0.001). There was a significant decrease of eotaxin positive cells in OVA-M (9.29 ± 0.86) and in OVA-D (7.03 ± 0.70) compared to OVA (*P* < 0.001). There were no differences between OVA-D and OVA-M groups.

### 3.3. Measurements of RANTES Expression

**Figure 3:**
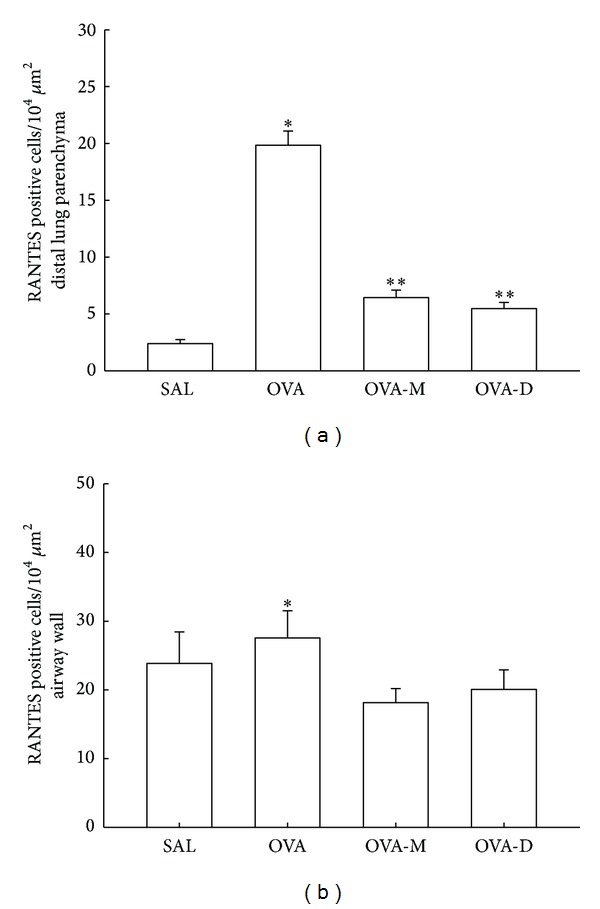
(a) Mean and SEM values of RANTES positive cells in distal lung of GP that previously inhaled normal saline or ovalbumin and after the 4th inhalation GP were treated with montelukast (OVA-M group) and dexamethasone (OVA-D group). *P < 0.001 compared to the other groups.^  ∗∗^ *P* < 0.001 compared to SAL group. (b) Mean and SEM values of RANTES positive cells in airway wall of GP that previously inhaled normal saline or ovalbumin and after the 4th inhalation GP were treated with montelukast (OVA-M group) and dexamethasone (OVA-D group). *P < 0.05 compared to the other groups.

The RANTES expression in distal lung parenchyma is shown in Figure 3(a). There was a significant increase in RANTES expression (positive cells/10^4^ *μ*m^2^) in OVA group (19.84 ± 1.25) compared to control (SAL group: 2.40 ± 0.35, *P* < 0.001). There was a significant decrease of RANTES positive cells in OVA-M (6.44 ± 0.66) and in OVA-D (5.47 ± 0.54) compared to OVA (*P* < 0.001). There were no differences between OVA-D and OVA-M groups.

Figure 3(b) shows RANTES expression in airway walls (positive cells/10^4^ *μ*m^2^). There was a significant increase in RANTES expression in OVA group (27.56 ± 1.83) compared to control (SAL group: 18.88 ± 1.31, *P* < 0.05). There was a significant decrease of RANTES positive cells in OVA-M (18.14 ± 1.30) and in OVA-D (20.05 ± 1.24) compared to OVA (*P* < 0.05). There were no differences between OVA-D and OVA-M groups.

### 3.4. Measurements of IGF-I

**Figure 4:**
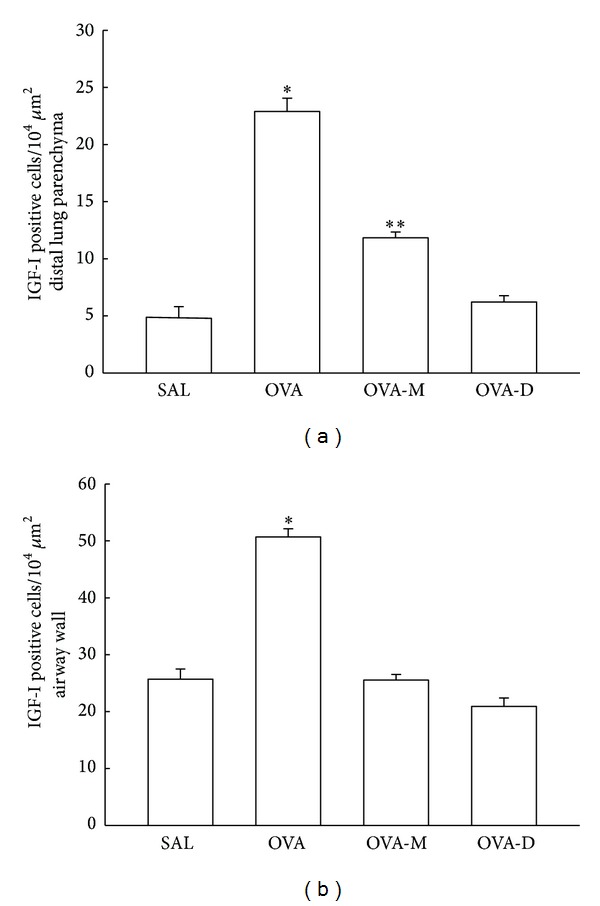
(a) Mean and SEM values of IGF-I positive cells in distal lung of GP that previously inhaled normal saline or ovalbumin, and after the 4th inhalation GP were treated with montelukast (OVA-M group) and dexamethasone (OVA-D group). *P < 0.001 compared to the other groups. **P < 0.001 compared to SAL and OVA-D groups. (b) Mean and SEM values of IGF-I positive cells in airway wall of GP that previously inhaled normal saline or ovalbumin, and after the 4th inhalation, GP were treated with montelukast (OVA-M group) and dexamethasone (OVA-D group). *P < 0.001 compared to the other groups.

Considering IGF-I expression in inflammatory cells (Figure 4(a)), we observed a significant increase in the number of IGF-I positive cells (10^4^ *μ*m^2^) in distal lung parenchyma in OVA group (22.89 ± 1.16) compared to control (SAL group: 4.87 ± 0.93, *P* < 0.001). There was a decrease of IGF-I positive cells in OVA-M (11.83 ± 0.51) and OVA-D (6.21 ± 0.56) compared to OVA (*P* < 0.001). There was a significant reduction of IGF-I positive cells in OVA-D compared to OVA-M (*P* < 0.001). There were no differences between OVA-D compared to SAL group.

Figure 4(b) presents the mean and SEM values of IGF-I positive cells (10^4^ *μ*m^2^) in airway walls. We observed a significant increase in expression of IGF-I positive cells in OVA group (50.70 ± 1.43) compared to control (SAL group: 25.70 ± 1.79). There was a decrease of IGF-I positive cells in OVA-M (25.53 ± 1.36) and OVA-D (20.90 ± 4.35) compared to OVA (*P* < 0.001). There were no differences between OVA-M and OVA-D groups.

### 3.5. Measurements of Fibronectin Positive Cells

**Figure 5:**
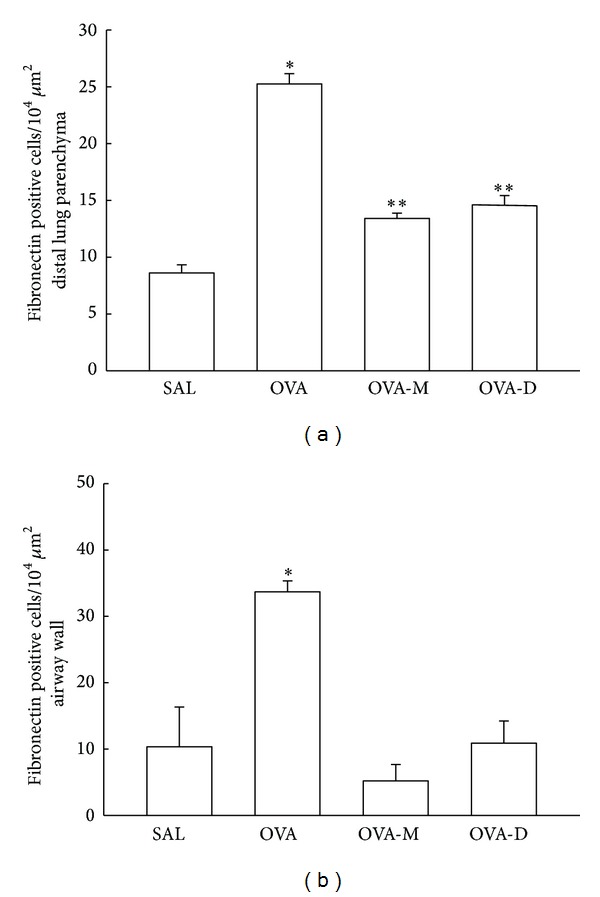
(a) Mean and SEM values of fibronectin positive cells in distal lung of GP that previously inhaled with normal saline or ovalbumin, and after the 4th inhalation, GP were treated with montelukast (OVA-M group) and dexamethasone (OVA-D group). *P < 0.001 compared to the other groups. **P < 0.001 compared to SAL group. (b) Mean and SEM values of Fibronectin positive cells in airway wall of GP that previously inhaled normal saline or ovalbumin, and after the 4th inhalation, GP were treated with montelukast (OVA-M group) and dexamethasone (OVA-D group). *P < 0.001 compared to the other groups.

Figure 5(a) shows the number of fibronectin positive cells (10^4^ *μ*m^2^) in distal lung parenchyma. We observed a significant increase in the number of fibronectin positive cells in OVA group (25.25 ± 0.90) compared to control (SAL group: 8.61 ± 0.70, *P* < 0.001). There was a significant decrease in the number of fibronectin positive cells in OVA-M (13.41 ± 0.47) and OVA-D (14.61 ± 0.82), compared to OVA group (*P* < 0.001). There were no significant differences between OVA-D and OVA-M groups.

Figure 5(b) shows the number of fibronectin positive cells (10^4^ *μ*m^2^) in airway walls. We observed a significant increase in the number of fibronectin positive cells in OVA group (33.69 ± 1.66) compared to control (SAL group: 10.35 ± 5.98, *P* < 0.001). There was a significant decrease in the number of fibronectin positive cells in OVA-M (5.21 ± 2.47) and OVA-D (10.88 ± 3.33) animals compared to OVA group (*P* < 0.001). There were no significant differences between OVA-D and OVA-M groups.

### 3.6. Measurements of NF-*κ*B

**Figure 6:**
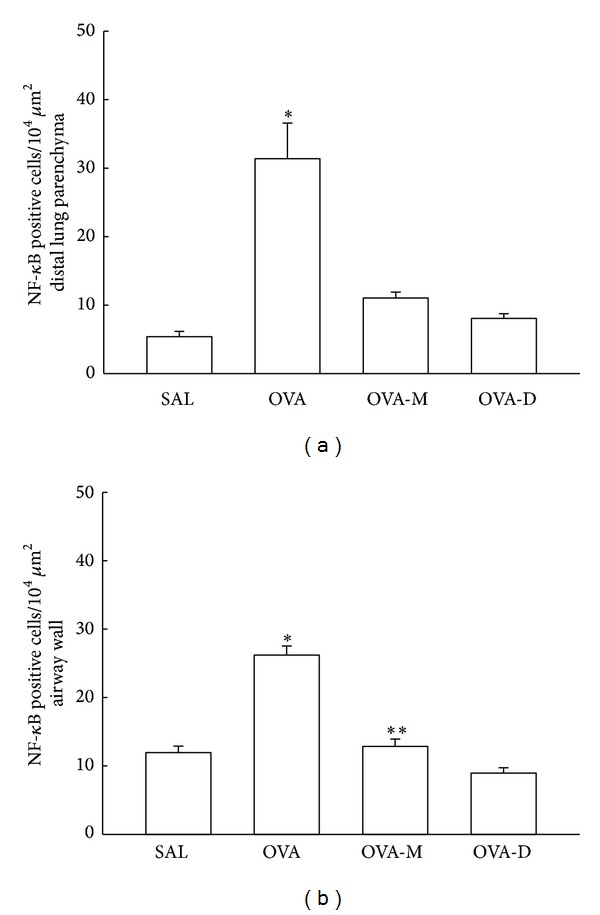
(a) Mean and SEM values of NF-*κ*B positive cells in distal lung of GP that previously inhaled normal saline or ovalbumin, and after the 4th inhalation, GP were treated with montelukast (OVA-M group) and dexamethasone (OVA-D group). *P < 0.001 compared to the other groups. (b) Mean and SEM values of NF-*κ*B positive cells in airway wall of GP that previously inhaled normal saline or ovalbumin, and after the 4th inhalation, GP were treated with montelukast (OVA-M group) and dexamethasone (OVA-D group). *P < 0.05 compared to the other groups. **P < 0.05 compared to OVA-D group.

Figure 6(a) shows the NF-*κ*B expression in distal parenchyma. We observed a significant increase in the number of NF-*κ*B (positive cells/10^4^ *μ*m^2^) in OVA group (31.38 ± 5.21) compared to control (SAL group: 5.38 ± 0.79, *P* < 0.001). There was a significant decrease in the number of NF-*κ*B positive cells in OVA-M (11.04 ± 0.86) and OVA-D (8.06 ± 0.70) compared to OVA group (*P* < 0.001). There were no differences between OVA-M and OVA-D groups.

Figure 6(b) shows the NF-*κ*B expression in airway walls. We observed a significant increase in the number of NF-*κ*B (positive cells/10^4^ *μ*m^2^) in OVA group (26.20 ± 1.34) compared to control (SAL group: 11.95 ± 0.96, *P* < 0.05). There was a significant decrease in the number of NF-*κ*B positive cells in OVA-M (12.84 ± 1.09) and OVA-D (8.95 ± 0.78) compared to OVA group (*P* < 0.05). Nevertheless, dexamethasone group (OVA-D) was significantly reduced in comparison to montelukast group (OVA-M). There were no differences between OVA-M and OVA-D groups when compared to SAL group.

### 3.7. Representative Photomicrographs of Airway Walls

**Figure 7:**
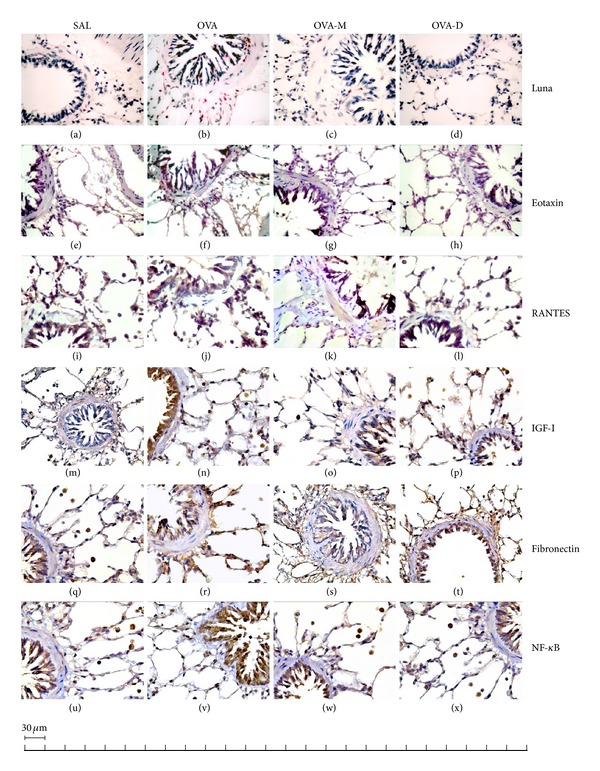
Representative photomicrographs showing guinea pig airway wall samples obtained from controls ((a), (e), (i), (m), (q), and (u)), OVA-exposed ((b), (f), (j), (n), (r), and (v)), OVA-exposed MK treated ((c), (g), (k), (o), (s), and (w)), and OVA-exposed D treated ((d), (h), (l), (p), (t), and (x)) stained with LUNA ((a) to (d)—×1000) and immunohistochemistry for eotaxin detection ((e) to (h)—×1000), RANTES detection ((i) to (l)—×1000), IGF-I detection ((m) to (p)—×1000), fibronectin detection ((q) to (t)—×1000), and NF-*κ*B detection ((u) to (x)). Control groups show a scarce number of eosinophils, eotaxinpositive cells, RANTES positive cells, IGF-I positive cells, fibronectin positive cells, and NF-*κ*B positive cells. In contrast, the airway wall of OVA-exposed animals shows intense eosinophilic infiltration (b), a large number of eotaxin positive cells (f), RANTES positive inflammatory cells (j), IGF-I positive inflammatory cells (n), fibronectin positive inflammatory cells (r), and NF-*κ*B positive inflammatory cells. Both treatments (MK and D) in ovalbumin-exposed animals reduced all these parameters.

Figure 7 shows representative photomicrographs of guinea pigs airway wall samples. We observed a significant increase in the number of eosinophils, eotaxin positive cells, RANTES positive cells, IGF-I positive cells, fibronectin positive cells, and NF-*κ*B positive cells in OVA group compared to control (SAL group). There was a significant decrease in eosinophils, eotaxin positive cells, RANTES positive cells, IGF-I positive cells, fibronectin positive cells, and NK-*κ*B positive cells in OVA-M and OVA-D compared to OVA group.

### 3.8. Representative Photomicrographs of Distal Lung Parenchyma

**Figure 8:**
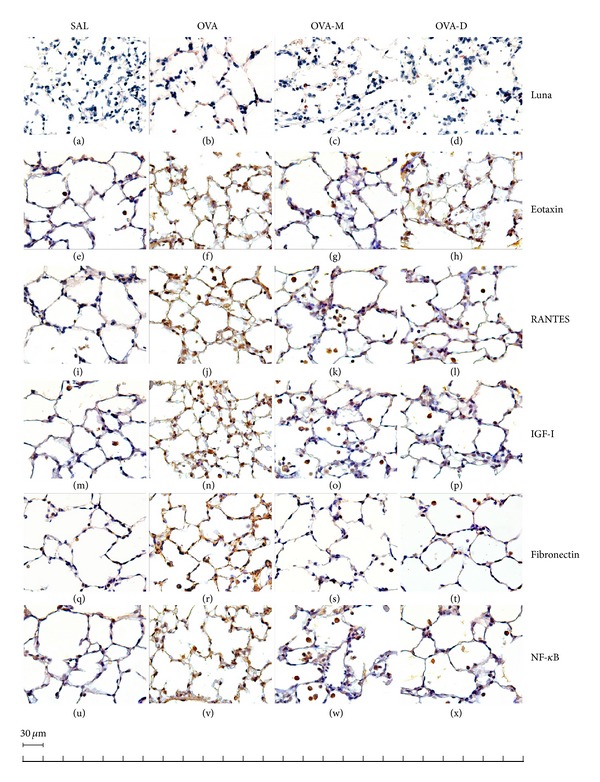
Representative photomicrographs of guinea pigs distal lung samples obtained from controls ((a), (e), (i), (m), (q), and (u)), OVA-exposed ((b), (f), (j), (n), (r), and (v)), OVA-exposed MK treated ((c), (g), (k), (o), (s), and (w)), and OVA-exposed D treated ((d), (h), (l), (p), (t), and (x)) stained with LUNA ((a) to (d)—×1000) and immunohistochemistry for eotaxin detection ((e) to (h)—×1000), RANTES detection ((i) to (l)—×1000), IGF-I detection ((m) to (p)—×1000), fibronectin detection ((q) to (t)—×1000), and NF-*κ*B detection ((u) to (x)). Control groups show a scarce number of eosinophils, eotaxin positive cells, RANTES positive cells, IGF-I positive cells, Fibronectin positive cells and NF-*κ*B positive cells. In contrast, the distal lung parenchyma of OVA-exposed animals shows intense eosinophilic infiltration (b), a large number of eotaxin positive cells (f), RANTES positive inflammatory cells (j), IGF-I positive inflammatory cells (n), fibronectin positive inflammatory cells (r), and NF-*κ*B positive inflammatory cells. Both treatments (MK and D) in ovalbumin-exposed animals reduced all these parameters.

Figure 8 presents representative photomicrographs of guinea pigs distal parenchyma samples. We observed a significant increase in the number of eosinophils, eotaxin positive cells, RANTES positive cells, IGF-I positive cells, fibronectin positive cells, and NF-*κ*B positive cells in OVA group compared to control (SAL group). There was a significant decrease in eosinophils, eotaxin positive cells, RANTES positive cells, IGF-I positive cells, fibronectin positive cells, and NK-*κ*B positive cells in OVA-M and OVA-D compared to OVA group.

It is important to mention that two more control groups were performed: saline groups treated either with montelukast (SAL-M) or dexamethasone (SAL-D). There were no statistical significant differences among the three control groups, so we showed only one of them.

## 4. Discussion 

Although corticosteroids are highly recommended as a first line therapy for asthma, it is important to highlight that montelukast can have a consistent benefit in controlling asthma symptoms and can be used as an alternative for those patients who have difficulties using inhaled corticosteroids (especially young children) or have some kind of intolerance with dexamethasone therapy [33]. Furthermore, a reduced adherence with inhalants for asthma compared to those therapies which are orally administrated has been addressed [34, 35].

To elucidate the role of antileukotrienes and corticosteroids in the control of changes in asthma remodeling, we evaluated the effects of montelukast or dexamethasone treatments on eosinophilic recruitment, including its activation by detecting eotaxin and RANTES positive cells in airways and lung parenchyma in guinea pigs (GP), with chronic allergic lung inflammation. Furthermore, to better comprehend the mechanisms involved in the remodeling process, we evaluated IGF-I and fibronectin in distal lung parenchyma and airway walls. Considering the importance of NF-*κ*B, one of the most important transcriptional factors involved in asthmatic responses, we also analyzed the expression of this protein in both pulmonary compartments.

We initiated these treatments only one day after the fourth inhalation, because in a previous study, using passive cutaneous anaphylaxis technique (PCA) [10], we observed an increase in specific immunoglobulin G1 (IgG1) homocytotropic anaphylactic antibodies in GP that received four inhalations with ovalbumin (at the end of the second week of this protocol).

Dexamethasone is considered a very potent anti-inflammatory therapy available for asthma. Its effects are probably related to a broad anti-inflammatory actions, including a decrease in airway infiltration of lymphocytes and eosinophils [36]. However, it is important to emphasize that one of the major challenges in using corticosteroids is related with their side effects [37], corroborating the need for better treatments that can improve the quality of life of asthmatic patients.

CysLTs play a major role in the pathophysiology of asthma, activating and recruiting some inflammatory cells, promoting airway remodeling, and increasing bronchial hyperreactivity and vascular permeability [38, 39].

Antileukotrienes are a new class of anti-inflammatory drugs, which includes, Zafirlukast, Montelukast, Pranlukast, and Zileuton. Montelukast is known to be a highly selective, pharmacological antagonist of type 1 cysteinyl leukotriene receptors (CysLT_1_Rs) which can recognize the cysteinyl leukotrienes (CysLTs) LTD_4_ and LTC_4_/LTE_4_ expressed on some inflammatory cells such as basophils, neutrophils, and lymphocytes and also on some structural cells such as fibroblasts, myofibroblasts, smooth muscle cells, and epithelial cells [38].

Since montelukast acts as a potent antagonist of the proinflammatory aspects of the CysLTs, it reduces asthmatic inflammation and airway resistance and also prevents bronchoconstriction, controlling asthma inflammation and improving inflammatory and pulmonary responses [40, 41].

Many studies in patients with asthma demonstrated a decrease in the number of eosinophils in the airways after treatment with corticosteroids [42]. Corticosteroids can cause the induction of eosinophils apoptosis by the mechanism known as programmed cell death, acting as a target therapy in asthmatic patients [43]. McMillan et al. [21] showed in mice chronically exposed to ovalbumin that chronic administration of budesonide was able to decrease airway hyperreactivity, as well as leukocyte infiltration, and decreased production of Th2 mediators such as interleukin 4 (IL-4), interleukin 12 (IL-12), and eotaxin-1.

Concerning eosinophil recruitment, we observed that both treatments tested were efficient in reducing this infiltration in distal lung and in airway walls.

Not only cysteinyl leukotrienes can act as a chemotactic for eosinophils, but also RANTES, eotaxin and eotaxin 2 are chemotactic substances that are involved during eosinophilic recruitment [44–46].

Eotaxin and RANTES are known as members of the C-C family of chemokines. The most important characteristic of the C-C chemokines is their facility to chemoattract and activate inflammatory leucocytes, particularly lymphocytes, monocytes, eosinophils, and basophils, and also some stromal cells such as endothelial and smooth muscle cells [47]. Another effect of eotaxins is their ability to cause immunoglobulin E(IgE)-independent degranulation of basophils [48].

A family of receptors mediates the activities of C-C chemokines; in general, any C-C chemokine can bind to several C-C receptors. Eotaxin is exceptional and binds only to the chemokine receptor type 3 (CCR3). Furthermore, eotaxin can chemoattract and activate eosinophils specifically, while RANTES are less specific to eosinophils, chemoattracting and activating other inflammatory cells as monocytes and lymphocytes [49].

Montelukast has mainly antieosinophil properties [50, 51]. This could be one of the possible explanations in our study suggesting why montelukast showed a better performance when compared to dexamethasone treatment while reducing eotaxin positive cells in distal parenchyma. Another possibility is that eotaxins are considered to be more specific than RANTES when activating eosinophils during the inflammation process.

A huge number of cells expressing eotaxin mRNA and protein in the bronchial mucosa area of atopic asthmatics compared with controls have been demonstrated by Ying et al. [52] and Mattoli et al. [53]. Furthermore, Ying et al. [52] showed CCR3 expression on local infiltrating eosinophils. These studies reinforce the theory that eotaxin, by the action of binding only to CCR3, plays a role in the specific recruitment of eosinophils to the asthmatic bronchial mucosa, a phenomenon which in turn regulates asthma severity.

In many asthmatic patients, the addition of anti- leukotriene can be a valuable approach for uncontrollable asthma regardless of treatment with inhaled corticosteroid despite the fact that little is known about its molecular mechanism.

Wu et al. [54] studying to the effect of a 3-day course of high-dose montelukast on mediators of airway inflammation induced by a single allergen challenge in sensitized mice showed that eotaxin protein and mRNA expression in the lung remained unchanged.

Uguccioni et al. [55] using pranlukast (another anti-leukotriene) in vitro, evaluated eosinophil transmigration across human umbilical vein endothelial cells in response to LTD4, eotaxin, RANTES, and platelet-activating factor (PAF). They showed that pranlukast did not modify eosinophil transmigration in response to eotaxin, RANTES, or PAF.

Nevertheless, in the present study, both treatments tested were efficient in reducing eotaxin positive cells in distal lung and in airway walls. Although montelukast treatment showed more efficiency in reducing eotaxin positive cells in distal lung parenchyma compared to dexamethasone treatment, both of them reduced these parameters in airway walls.

Some studies have shown the chemoattractant power activity of RANTES for eosinophils in airway allergic inflammation. Gonzalo et al. [56] showed that treatment with methylated RANTES (a CCL5 antagonist) decreased eosinophilia after antigen challenge by blocking the activation of RANTES and its binding to the receptor.

Regarding the evaluation of RANTES positive cells, we noted that both treatments have shown effectiveness either in distal lung parenchyma or in the airway wall contributing to a better control of asthma.

Lung remodeling and chronic inflammation are important characteristics of asthma. It has been shown that modulation of fibroblasts into myofibroblastic phenotype, with addition of particular contractile aspects, indispensable for connective tissue remodeling during the wound healing process in asthmatics airways [57]. Nonetheless, the connection between fibroblasts, myofibroblasts, and smooth muscle cells needs to be better investigated [58].

Fibroblasts are responsible for producing a wide array of extracellular matrix components like collagen, reticular and elastic fibers, laminin, fibronectin, hyaluronic acid, glycoproteins, and proteoglycans amorphous extracellular substance [59, 60]. Nam et al. [61] showed that primary bronchial fibroblasts were susceptible to mechanical stimuli during differentiation into myofibroblasts. Furthermore, they have addressed that fibroblasts from asthmatics showed higher potential for tissue fibrosis when compared to control group.

Myofibroblasts are known to be contractile cells with biochemical and morphologic aspects that stay somewhere between fibroblasts and smooth muscle cells [62, 63]. TGF-*β* is the major regulator that promotes myofibroblast differentiation through its capacity to accumulate intracellular contractile proteins, fibronectin containing extra type III domain A (EDAcFN), and collagen density [57].

Through inflammatory mesenchymal and epithelial cells, a multimeric form of cellular fibronectin is produced, and it is deposited in the fibrils of the extracellular matrix, containing variable portions of the extra type III domain A and B (EDA and EDB) sequences [64]. These domains are known to play an important role in the contribution of EDAcFN to fibroblast activation [65] and wound healing process [66].

Fibronectin matrix formation is a dynamic, cell-dependent process that is tightly regulated. The loss of this regulation promotes an increase in the deposition of fibronectin and other extracellular matrix (ECM) molecules in the subepithelial matrix in patients with asthma. A specific site of fibronectin modulates cell contractility, collagen gel contraction, and cell migration. Furthermore, the increased or inappropriate exposure of this site in matrix fibronectin may lead to abnormal tissue remodeling by enhancing and/or prolonging cell contractility and by altering the rate of reepithelialization. The development of new strategies to control the extent and duration of the exposure of cells to matrix-specific epitopes can supply a functional method to structure normal tissue remodeling in asthma [6].

The cysteinyl leukotriene LTD4 causes a potentiation of fibronectin-induced migration of human lung fibroblasts [67], contributing to the airway remodeling process. Furthermore, Tokuriki et al. [68] observed that the leukotriene D_4_ acts as a precipitating factor during the remodeling mediated by endothelin-1, and antileukotrienes have a role in preventing aberrant extracellular matrix degradation. An experimental mice model of asthma showed that the antileukotrienes inhibit the process of airway remodeling, including the influx of eosinophils into the lungs, eosinophil degranulation, the release of Th2 cytokines, hyperplasia of mucous glands, hypersecretion mucus, hyperplasia of smooth muscle cells, collagen deposition, and pulmonary fibrosis [69].

In this study, we observed an increase in fibronectin positive cells in chronic allergic lung inflammation model. Both treatments tested were able to decrease the number of fibronectin positive cells in distal lung parenchyma and in distal airway walls.

Fibrosis and asthma are highly linked by the increased deposition of extracellular matrix at specific places in the airway wall. Growth factors may be considered during asthma remodeling. Increased expression of TGF-*β* [70] has been shown in lung fibrosis and plays an important role in the excessive matrix deposition and also in cell proliferation. IGF-I is known to be a highly potent mitogen for fibroblasts and smooth muscle cells, acting on fibroblasts as a progression factor pushing the cells from a G1 phase to mitosis in the cell cycle, activating them to make the collagen synthesis [71]. It is important to emphasize that collagen deposition at the lamina reticularis contributes to the remodeling process, an asthma characteristic, enhancing the role that IGF-I can play as an important mediator of inflammation and remodeling in asthmatic airways.

Muz et al. [72] showed that airway collagen deposition/fibrosis was reduced after montelukast treatment, suggesting that cysteinyl-leukotrienes have an important role in collagen deposition/lung fibrosis. In addition, Henderson Jr. et al. [73] showed that montelukast could significantly reduce airway remodeling in a mouse model of chronic asthma, addressing montelukast effect in some aspects of the remodeling process.

Veraldi et al. [5] showed that insulin-like growth factor binding protein-3 IGFBP-3 levels are increased in bronchoalveolar lavage (BAL) fluid of asthmatic patients 48 hours after allergen challenge, suggesting that this is the most important protein that IGF binds, developing airway remodeling in asthma through this modulation. Considering this, the creation of an IGFBP-3 antagonist could be a promise therapeutic target for asthma treatment.

Rajah et al. [74] using cultured airway smooth muscle cells addressed the synergism between IGF and inflammatory agents such as leukotriene D_4_ and interleukin 1-beta, showing that these agents induce the secretion of an IGFBP protease which cleaves the IGFBPs secreted by airway smooth cells. This event allows IGF to stimulate proliferation, emphasizing that IGFBP proteases act as a critical element in asthma and other diseases. Rajah et al. [75] also reported that antimatrix metalloproteinase (MMP-1) acts as an IGFBP protease induced by leukotrienes that plays an important role in modulating IGF action in airway smooth muscle cells. Our findings showed that the anti-leukotriene montelukast reduced IGF levels in distal lung parenchyma and airway walls in experimental models of chronic allergic inflammation.

Hoshino et al. [76] have investigated the effects of inhaled corticosteroids in asthmatic patients. They observed a significant decrease in thickness of the subepithelial collagen and also an important reduction in the number of fibroblasts and the expression of IGF-I, suggesting that the decrease in collagen thickness after the treatment with inhaled corticosteroid could be a result of blocking the transcription of the IGF-I gene. In our study, we observed a decrease in IGF with both treatments. However, dexamethasone treatment was more efficient in reducing IGF-I positive cells in distal parenchyma when compared to montelukast treatment.

Corticosteroids are known to play an important role in the therapy of a large number of diseases, especially diseases of the gastrointestinal tract and liver [77]. Gayan-Ramirez et al. [78] showed that corticosteroid treatment in rats was highly linked with a reduction in IGF-I serum levels mainly after triamcinolone treatment that resulted from a decrease in IGF-I manifestation in the liver.

Our findings showed that dexamethasone was more efficient when compared to montelukast treatment in reducing the expression of IGF-I in distal lung parenchyma. This could be explained since this growth factor is synthesized and released by the liver and can be mediated by the action of corticosteroids [79].

Further studies are needed to ascertain the molecular mechanisms involving IGF-I and the current therapies.

NF-*κ*B is a transcription factor, and it plays an important role in expression of lots of proinflammatory genes, which leads to the synthesis of growth factors, adhesion molecules, and chemokines [80].

Lin et al. [81] showed that total lung extracts from brownNorway rats exhibit enhanced NF-*κ*B activity after the ovalbumin (OVA) sensitization and challenge model. There is also a relation between NF-*κ*B and nitric oxide (NO). When inducible nitric oxide synthase (iNOS) is activated, it generates a high concentration of NO through the activation of some inducible nuclear factors, including the NF-*κ*B [82].

Our results showed a higher efficacy of corticosteroids compared to montelukast treatment in reducing NF-*κ*B in airway walls. This can be explained by the broad mechanism that corticosteroids play, by switching off the expression of multiple genes, including adhesion molecules and chemokines [83].

NF-*κ*B activity is highly regulated by its interaction with I*κ*B proteins. One possible explanation to corticosteroids higher efficacy in reducing NF-*κ*B is that they can be involved in regulating the expression of I*κ*B proteins. Another possibility is that NF-*κ*B is regulated during the interaction with other coactivators, such as transcriptional co-activator proteins (CBP/p300), which are highly known to interact with NF-*κ*B promoting transcription [83].

Arachidonate 5-lipoxygenase (ALOX5) is an enzyme that synthetizes the cysteinyl leukotrienes LTC_4_, LTD_4_, LTE_4_, and LTB_4_. ALOX5 inhibition can be associated with an improvement in asthma outcome, and it is known that the ALOX5 gene promoter has binding-sites for some transcription factors, including NF-*κ*B. However, not all asthmatic patients will respond to antileukotrienes since those who have mutant alleles at the ALOX-5 should not have clinical benefits [84]. This could be a possible explanation of the relative lack of response by montelukast treatment in reducing NF-*κ*B in airway walls.

Considerable attention has been given to the signaling pathways related with the NF-*κ*B activation. These signaling protein molecules can be, in the near future, a potential therapeutic target to block NF-*κ*B activation, interrupting the asthma process. Further studies are needed to better understand how these molecules interact with each other [85].

Despite the fact that there is current evidence of the importance of these treatments on airways regarding the control of main morphofunctional alterations present on asthma, the present study enlightens that isolated treatment with these drugs was chiefly capable of controlling also distal lung parenchyma alterations.

It is worthy to mention that, in the present study, all of the drugs were systemically administered, not through inhalation that probably contributed to the efficacy of the treatment on distal lung parenchyma.

Despite all the scientific and ethical care involved in the insertion of a new form of treatment for any disease, only after some time of use we can adequately characterize its efficacy's spectrum. This motivates the need for practical applications that help people. In other words, translational medicine, research that aims to move “from bench to bedside” or from laboratory experiments through clinical trials to point-of-care patient applications or dissemination to population-based community interventions. Thus, this study used and experimental model of chronic allergic inflammation to better understand the mechanisms involved in the pathophysiology of asthma. We observed important results on the relation of the available treatments for allergic diseases and the immune response on the asthmatic patient. Our goal is a continuous feedback loop to accelerate the translation of data into knowledge from the basic sciences into the development of new treatments, translating the findings from clinical trials into everyday practice.

## 5. Conclusion

In this animal model, both corticosteroid and montelukast treatments were effective in the control of the inflammatory response in distal lung parenchyma and distal airway walls. But it is noteworthy to emphasize the contribution of montelukast in the resolution of some inflammatory and remodeling aspects in this experimental model, mainly in the control of distal lung parenchyma functional and histopathological alterations.

In conclusion, asthma is a chronic disease and corticosteroids are considered to be a gold standard medical therapy. However, since corticosteroids can have a wide range of side effects, it is essential that studies of therapeutic efficacy consider alternative treatments. Antileukotrienes are an orally, safe administered, nonsteroidal therapy for treating mild persistent asthma, especially in young children. They are an option for the treatment of asthma, based on many of its biological effects described above.

Therefore, our data contributes to a better understanding of the inflammatory and remodeling processes in both treatments in this experimental model of asthma and may assist researchers to further studies based on their expected outcomes.
